# Autonomous UAV Target Search Method Based on Lightweight YOLOv8n and Coverage Path Planning

**DOI:** 10.3390/s26103247

**Published:** 2026-05-20

**Authors:** Haoyan Duan, Zhenhua Wang, Mengtong Li, Zhenbo He, Haoxuan Zhang

**Affiliations:** 1 School of Artificial Intelligence, China University of Geosciences, Beijing 100083, China; m15222660518@163.com (H.D.); 1004243128@email.cugb.edu.cn (Z.H.); 1004243117@email.cugb.edu.cn (H.Z.); 2 School of Computer Science, Peking University, Beijing 100871, China; morten.li@outlook.com

**Keywords:** UAV, object detection, path planning, breadth-first search, greedy algorithm

## Abstract

**Highlights:**

**What are the main findings?**

- A lightweight YOLOv8n model is proposed via channel pruning and INT8 quantization, reducing inference latency on a Raspberry Pi 4B to 83 ms while maintaining 84.6% mAP@0.5.
- The grid-greedy-BFS (GGB) hybrid coverage path planning algorithm achieves 98.7% area coverage with a 83.8% time saving compared to pure BFS.

**What are the implications of the main findings?**

- The method enables real-time autonomous target search on resource-constrained UAV platforms.
- The hybrid planning strategy balances coverage completeness and search efficiency, supporting dynamic target replanning in unknown environments.

**Abstract:**

Unmanned aerial vehicles (UAVs) have wide application prospects in disaster search and rescue, ecological monitoring and environmental inspection tasks, where target search is a key link to realize autonomous task execution. UAVs often face challenges related to limited onboard computational resources and inefficient environmental coverage when used for target search. To address these issues, this paper proposes an autonomous search method for UAVs based on combined lightweight target detection and coverage path planning. In this method, the target search task was decomposed into two core parts: target recognition and path planning. Firstly, in terms of target recognition, the YOLOv8n model was subjected to channel pruning and INT8 quantization to reduce its computational complexity, while HSV space data augmentation was incorporated to enhance recognition robustness in complex environments. Secondly, path planning was formulated as a dual-layer task comprising “spatial coverage + target confirmation.” A grid-based search environment model was constructed, and a coverage path planning strategy was put forward that integrated breadth-first search (BFS) with local greedy optimization to achieve efficient traversal of predefined search areas. Simultaneously, the A* algorithm was employed for path backtracking to cover omitted regions. Finally, a simulation platform for UAV target search was built to validate the recognition performance and search efficiency of the proposed method. The experimental results demonstrated that the proposed method significantly improved the UAV target search efficiency and reduced the path redundancy while ensuring the recognition accuracy, thereby offering an effective solution for autonomous UAV search on resource-constrained embedded platforms.

## 1. Introduction

In recent years, unmanned aerial vehicles (UAVs) have been widely used in emergency rescue and other fields [1,2,3,4], and their autonomous ability is highly dependent on two core technologies [5,6,7]: target recognition and efficient path planning in complex environments. In the “14th Five-Year Plan” for civil aviation development, it is pointed out that it is necessary to innovate within the ecology of the UAV industry [8] and conduct operation scenario-oriented research on operation theory, risk assessment and technical verification based on operation risk analysis. UAVs are mostly used as camera modules in daily life, and existing algorithms are largely designed for structured environments and offer limited adaptability to dynamic target search, meaning that UAVs cannot be an independent unit for problem solving. In addition, the target recognition accuracy of UAVs may be lowered by illumination mutation, color distortion and occlusion in real life, and it is difficult for them to achieve a full-coverage cruise and dynamically respond to new tasks in an unknown environment, posing serious challenges for research into UAV target search algorithms [9,10].

For target recognition, UAV onboard platforms generally have limitations concerning their computational resources and power consumption, which cause trouble during the direct deployment of large-scale deep neural networks. The YOLO series models achieve a favorable balance between speed and accuracy through their end-to-end single-stage detection architecture [11], while the standard YOLOv8n model exhibits an inference latency of 125–200 ms on embedded platforms such as the Raspberry Pi 4B, thereby failing to meet real-time processing demands. Existing studies have improved the inference speed of the YOLO series on embedded platforms to some extent through model pruning and quantization techniques [12,13,14], though a critical challenge remains in minimizing accuracy degradation while maintaining model robustness against varying illumination conditions and color interference during this process [15]. Moreover, current methods predominantly focus on model optimization in specific scenarios and do not sufficiently account for the communication reliability associated with transmitting perception results [16,17,18].

Furthermore, the data quality of UAV visual recognition and the reliability of subsequent detection may be affected directly in complex open environments. The research on sensors for specialized unmanned ground vehicles by Nowakowski et al. noted that the operational environment can alter a sensor’s effective detection capabilities; factors such as lighting, rainfall, fog, dust, temperature fluctuations and surface reflectivity can all lead to increased measurement noise, reduced effective range or degradation of target edge information [19]. This conclusion also provides valuable insight for visual search using low-altitude UAVs: RGB cameras are prone to underexposure, motion blur, color drift and partial occlusion when UAVs fly over forests, grasslands or in the shadows of buildings, or in rainy or foggy conditions, which then reduce the confidence and localization accuracy of target detection models. Meanwhile, Li et al. pointed out that in UAV images, object detection occlusion occurs more frequently than in natural scenes; occlusion can result in feature confusion and local object clustering, causing general-purpose detectors to produce false negatives and false positives in UAV images [20]. Therefore, to solve the visual recognition problems, this paper not only introduces data augmentation in the HSV color space but also incorporates supplementary experiments involving low-light conditions, reflections, light rain and fog, complex textured backgrounds and occlusion scenarios in its conclusion, which explicitly highlight the limitations of relying solely on RGB perception in adverse environments, thereby providing references for future research into visual–infrared or multi-sensor fusion.

For path planning, coverage path planning (CPP) can be considered the core part of the search task. The traditional breadth-first search (BFS) algorithms have advantages in traversal completeness, but disadvantages in excessive path redundancy; conversely, greedy algorithms gain high efficiency, but critical areas are likely overlooked [21,22]. Although some scholars have proposed hybrid algorithms, there are few experimental verification algorithms combining target detection confidence for dynamic task planning [23,24]. In addition, UAVs have a high vulnerability to battery limitations, network disruptions and unstable communication links, which elevates bit-error rates, increases transmission delays and ultimately undermines the practicability of such systems [7,25,26,27]. Therefore, we incorporate data statistics on serial data transmission into the experiments and include path deviations caused by wind, light rain and fog, gusts and complex terrain as additional evaluation criteria.

In view of the above problems, this paper proposes a comprehensive algorithm framework for UAV target recognition and path planning in complex environments, which mainly includes: (1) implementing model lightweighting for YOLOv8n through channel pruning and INT8 quantization to accelerate inference speed on the Raspberry Pi 4B, and enhancing model robustness by introducing HSV color-space data augmentation so as to meet real-time processing requirements in complex color scenarios. (2) achieving rapid full coverage with high efficiency and strong dynamic target replanning capacity by integrating the local rapid optimization capability of greedy algorithms with the global coverage completeness of breadth-first search.

Finally, simulation and experimental verification were conducted within the preconfigured simulated field environment. The results show that the proposed algorithm performs well under a variety of different interference conditions, especially in the wild-animal search scene; the proposed algorithm has a maximum mAP@0.5 of 84.6%, an mAP@0.5:0.95 of 56.7% and a search coverage of 98.7%, significantly surpassing current methods.

## 2. Related Work

UAV targeting comprehensively covers issues of environment perception, path planning and decision control. Traditional research methods usually optimize target recognition and path planning as independent modules, lacking system-level collaborative design [9,28].

For object recognition, early studies mainly relied on methods combining hand-designed feature extractors (such as SIFT, HOG) with classical machine learning classifiers (such as SVM) [29]. These methods have achieved certain achievements in specific scenes, but obviously have insufficient generalization ability and robustness in the complex conditions frequently faced by UAV platforms, such as those with viewpoint changes, illumination interference and diverse target scales [30,31]. With the rise of deep learning technology, object detection algorithms based on convolutional neural networks have attracted more and more attention, among which the YOLO series achieves a good balance between detection speed and accuracy due to its end-to-end single-stage detection architecture, thereby making it especially suitable for resource-constrained platforms aboard UAVs [11].

In terms of path planning, traditional methods are mainly divided into two categories: classical algorithms and heuristic algorithms. Classical algorithms include the A* algorithm, Dijkstra algorithm, artificial potential field method, etc., which function steadily in static environments but have difficulties in dealing with dynamically changing task requirements. Heuristic algorithms such as genetic algorithms, the particle swarm optimization algorithm, the ant colony algorithm and so on are widely used in path optimization in complex environments, but have the problems of slow convergence speeds and a high likelihood of falling into local optimum [5,24,32]. As a core component of search tasks, full-coverage path planning is traditionally addressed by algorithms like BFS, which ensures exhaustive coverage at the cost of low efficiency, and greedy algorithms, which prioritize speed but often miss critical areas [22]. According to research in the IEEE journal, in uncertain environments, the communication constraints, target dynamics and task collaboration have to be taken into consideration during the coverage search of a UAV swarm, and it is difficult for the traditional methods to meet these requirements at the same time [33,34].

To solve the above problems, this paper proposes a UAV target search algorithm framework based on two core theories: lightweight deep learning theory and full-coverage path planning theory.

Lightweight deep learning theory provides a technical path for deploying complex vision models on resource-constrained UAV platforms. By removing redundant channels or connections in the neural network, model pruning can significantly decrease the amount of computation while maintaining model performance. Model quantization transforms high-precision floating-point parameters into low-bit integer representations, which greatly lowers the model’s size and accelerates inference [12,13,35]. Research by Soumyalatha et al. demonstrates that the combination of pruning and INT8 quantization technology reduces the parameters of the YOLOv3 model by 80.39% and improves the inference speed by 22.72% [13]. Considering the selection of YOLO series models, YOLOv8n provides an all-round improvement in performance compared with YOLOv5n. On the premise of ensuring a similar model size, the data of the YOLOv8n model trained on COCO show that its mAP@0.5:0.95 can be improved by about 10% compared with YOLOv5n. The results above lay a theoretical foundation for airborne real-time target detection.

The theory of full-coverage path planning provides a mathematical framework for the spatial traversal strategy of UAV search missions [5,36]. The full-coverage path planning problem can be modeled as finding an optimal path that can visit all passable grids in a given area. The BFS algorithm guarantees the completeness of the traversal from the perspective of graph theory, while the greedy algorithm pursues the local optimal solution from the perspective of optimization. The hybrid algorithm integrating the BFS and greedy algorithms enhances search efficiency while maintaining coverage [22]. At the same time, the target recognition module performs target comparison in real time during flight, so that the data of the two modules can be coordinated to ensure dynamic replanning ability. In the review of Sheltami et al., the technical classification system of UAV path planning has been systematically sorted out, and it was pointed out that about 30% of previous studies were involved in classical methods, 29% in metaheuristic methods, 18% in AI methods and 23% in hybrid methods [23]. Rahman et al. further emphasized that multi-UAV cooperative path planning posed requirements for designing intelligent algorithms with real-time replanning abilities in complex scenarios [24].

Compared to traditional single-path search methods, the GGB algorithm shifts its design focus from “shortest reach” to “efficient coverage”. In recent years, hybrid heuristic, multi-drone collaborative and learning-based methods have emerged as leading approaches in coverage path planning. The hybrid heuristic method typically improves coverage efficiency in complex environments through region decomposition and local optimization; the multi-drone method expands the search scope by dividing tasks; and the learning-based method emphasizes adaptability to dynamic environments. However, these methods often involve high system complexity, training costs or communication and coordination overheads, making it difficult for them to directly adapt to resource-constrained single-drone platforms. In contrast, the GGB algorithm employs lightweight grid modeling and combines a direction-priority local greedy strategy with BFS reachability checking and a missed-point recovery mechanism, which enhances coverage completeness and reduces path redundancy without relying on iterative training or multi-agent coordination. The experiment results showed that the GGB algorithm achieved a coverage rate of 98.7% in a simulated environment with a path planning time of 0.31 ms, indicating a high balance between real-time performance and coverage effectiveness, though the algorithm remains a lightweight hybrid heuristic framework and there is still room for improvement in terms of global optimality, adaptation to 3D terrain, energy consumption constraint modeling and scalability to multiple drones.

## 3. Methodology

### 3.1. Task Modeling

In this paper, the UAV target search task is formalized as an optimization problem with dual layers: full space coverage and accurate target confirmation.

Firstly, the task environment was divided into a grid map of *M* × *N*, and the size of each grid matched the field of view of the UAV, which was initially set to 1 m × 1 m. The map state could be represented as a matrix *Zxy*, where the element *m* took the value 1 (visited), 0 (none visited) or −1 (obstacle) [20]. The goal of the full-coverage layer was to plan a path so that the UAV could traverse all the obstacle-free grids within the energy constraint to complete the initial search. Upon receiving high-confidence suspected target indications from the detection module, the target confirmation layer was activated, prompting the UAV to suspend its current coverage task and formulate a localized path for secondary detailed inspection of the designated area. This modeling method separates “wide-area search” from “fixed-point confirmation” and takes into account both search breadth and mission reliability.

### 3.2. Model Architecture

**Figure 1:**
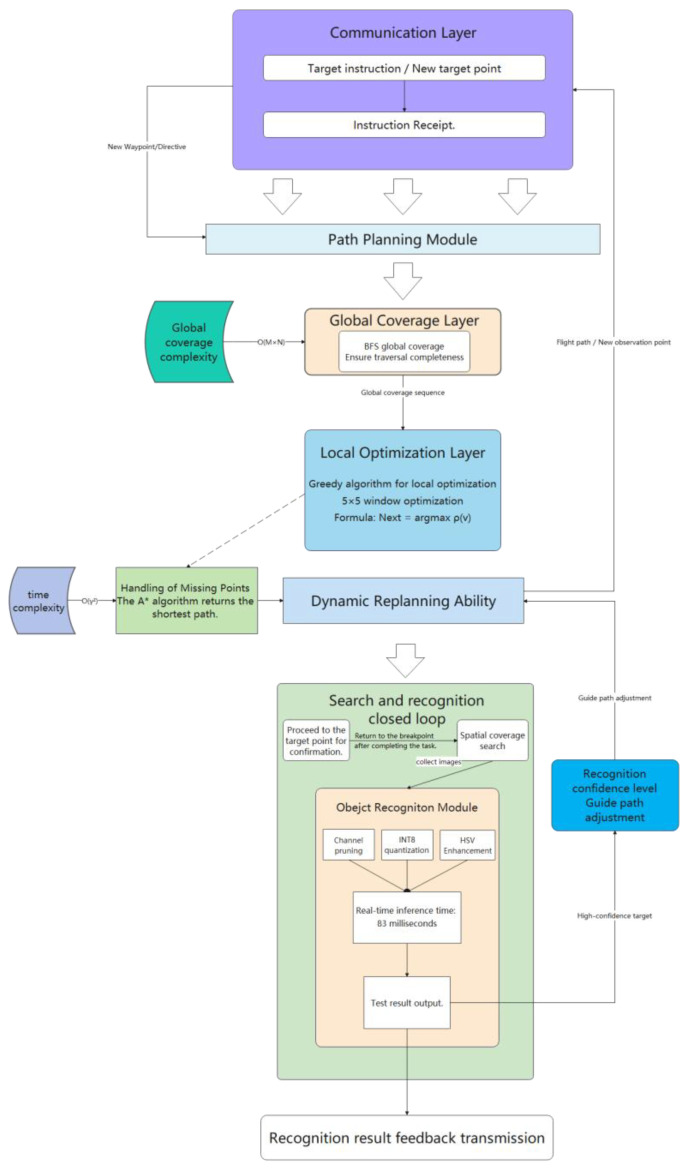
Flowchart for UAV Autonomous Search Methodology.

Based on the ideas proposed by the above task modeling, this paper constructs a three-in-one algorithm architecture of “identification–transmission–planning”. The overall framework is shown in Figure 1.

The Algorithm 1 implements YOLO-based real-time object detection and sends the detection results to external devices through the serial port. Initially, the system initialized serial communication, the YOLO detection model, the camera and the display device. Then, it entered the main loop: The image was obtained from the camera and input into the detector for object recognition. When the target was detected, the location information, category label and confidence of each target were extracted one by one, encapsulated as a data packet, and sent out through the serial port. In case no target was detected, an empty data packet was sent to indicate that there was no target at present.
**Algorithm 1:** Object Detection and Serial Transmission**1:****Initialize serial, detector, cam, disp****2:****while not terminated do****3:**   **img ← cam. read ()****4:**   **objs ← detector. detect (img)****5:**   **if objs is not empty then****6:**     **for each obj in objs do****7:**       **extract (x, y, w, h, label, score)****8:**       **data ← package (x, y, w, h, label, score)****9:**       **serial. send (data)****10:**     **end for****11:**   **else****12:**     **data ← package (0, 0, 0, 0, 0, 0)****13:**     **serial. send (data)****14:**   **end if****15:**   **disp. show(img)****16:****end while**

The Algorithm 2 was designed to form a lightweight and reliable transmission mechanism, so as to prevent the serial port delays or data transmission failures that might occur in environments with extreme vibration and strong electromagnetic interference. UART, a baud rate of 921,600 bps, an 8N1 format and a circular buffer were involved in the initial serial communication, with three retransmissions at most. Data packets were constructed by encapsulating the processed single-frame images, with a frame header, sequence number, timestamp, payload and a CRC16 checksum in each packet. Each packet was retransmitted up to three times. After each transmission, a 20-ms timeout timer was started to wait for an ACK or NACK from the receiver. If an ACK with a matching sequence number was received, the transmission was confirmed as being successful; if a NACK was received or there was no response within the timeout period, a retry was initiated. The receiver temporarily stored acknowledged packets in the circular buffer and verified the CRC. If the verification succeeded and the sequence numbers were consecutive, the planner’s target was updated; otherwise, the packet was discarded and a retransmission was requested. If CRC verification failed or ACK timeouts occurred for five consecutive frames, the planner no longer used the current potentially erroneous coordinates but retained the previous valid target coordinates to prevent erroneous coordinates from directly entering GGB replanning.
**Algorithm 2:** Reliable Serial Communication**1: initialize UART(S), seq ← 0** **2:  while system is running do** **3:    frame ← camera.read()** **4:    result ← D.detect(frame)** **5:    payload ← encode(result) if result is not empty else NULL_TARGET** **6:    packet ← build_packet(seq, timestamp, payload, CRC)** **7:    send packet through S and wait for ACK** **8:    if ACK is received then** **9:      P.update_target(payload)** **10:      seq ← (seq + 1) mod 256** **11:    else** **12:      P.use_last_valid_target()** **13:      if continuous failures exceed threshold then** **14:       P.switch_to_safe_mode()** **15:      end if** **16:    end if** **17:    log communication status** **18: end while**

The algorithm, through the aforementioned mechanism, ensured reliable transmission of target detection results from the perception module to the planning module even under adverse communication conditions such as vibrations and electromagnetic interference, effectively preventing abnormal UAV behavior caused by the loss or corruption of individual data packets.

This Algorithm 3 was designed to generate a feasible path in a grid environment with no-fly-zone constraints, and the path was compressed as follows: Firstly, the reachability test was used to determine whether all feasible areas could be covered from the starting point. An error was directly returned in case of any point being unreachable. Then, starting from the initial point, a traversal strategy based on direction priority was adopted to gradually expand the path and mark the visited nodes until no further progress could be made. For any remaining unvisited nodes, the BFS algorithm was utilized to identify the shortest paths from the current position to these nodes, which were then integrated into the main path to ensure comprehensive coverage of all reachable regions. After the coverage was completed, the path was compressed to remove redundant intermediate points and only the turning points were retained to reduce the path length and execution cost. Finally, the algorithm connected the current position back to the starting point through one BFS to form a closed path.
**Algorithm 3:** Path Search and Compression**1: if not isReachable (start, forbidden) then return error** **2: current ← start, path ← [start], visited[start] ← true** **3: while there exists unvisited reachable nodes do** **4:   next ← a valid neighboring node selected from directions** **5:   if next exists then** **6:     current ← next, visited[current] ← true, path ← path + current** **7:   else** **8:     break** **9:   end if** **10: end while** **11: for each unvisited node p do** 12:    path ← path + A*(current, p) **13:    current ← p** **14: end for** **15: path ← compressPath (path)** 16: path ← path + A* (current, start) **17: return path**

#### 3.2.1. Object Recognition Module

The target recognition module uses YOLOv8n as the basic model. Its core convolution formula is as follows. The symbols commonly used in this article and their meanings are summarized in Table 1.

(1) $$Y\left(i,j,k\right)=\sum_{c=1}^{C}\sum_{m=1}^{M}\sum_{n=1}^{N}X\left(i+m,j+n,c\right)\cdot W\left(m,n,c,k\right)+b_{k}.$$

The goal of the YOLO loss function is to minimize the difference between each predicted box and the true box. The formula is as follows:

(2) $$L=\lambda_{coord}\sum_{i=0}^{S^{2}}1_{obj}\left[(x_{i}-{\hat{x}}_{i}{)}^{2}+(y_{i}-{\hat{y}}_{i}{)}^{2}+(w_{i}-{\hat{w}}_{i}{)}^{2}+(h_{i}-{\hat{h}}_{i}{)}^{2}\right]c+\;\lambda_{noobj}\sum_{i=0}^{S^{2}}1_{noobj}\left[(C_{i}-{\hat{C}}_{i}{)}^{2}\right]+\sum_{i=0}^{S^{2}}1_{obj}\left[(p_{i}-{\hat{p}}_{i}{)}^{2}+(C_{i}-{\hat{C}}_{i}{)}^{2}\right].$$

In parallel, lightweight optimization was performed specifically for the Raspberry Pi 4B platform. Firstly, sparse training was carried out on the final C3 module to remove 40% of the redundant channels. After pruning, the mAP@0.5 loss of the model was controlled within 2%, which effectively reduced the computational complexity. Secondly, ONNX Runtime INT8 quantization technology was used. The model size was compressed from 6 MB to 4.2 MB by combining the two methods of pruning and quantization, and the recognition speed of a single image was improved from 48 ms/img to 26 ms/img. The overall model inference latency reached 83 ms, meeting the requirement for real-time onboard processing. Channel pruning and INT8 quantization were used to achieve lightweight compression. The loss of the pruned model is calculated as follows:

(3) $$\Delta mAP=\frac{mAP_{\mathrm{after}}-mAP_{\mathrm{before}}}{mAP_{\mathrm{before}}},$$

the inference speed improvement formula after quantization is

(4) $$Speedup=\frac{T_{before}}{T_{after}}.$$

In addition, the model further incorporated an HSV color-space data augmentation strategy to account for complex lighting conditions and color interference. Specifically, random adjustments were applied, including H-shift offsets within the range of [−15°, +15°], S-scale factors between [0.5, 1.5] and V-shift offsets within [−20, +20]. This approach emulated realistic illumination variations in practical scenarios and enhanced the model’s adaptability to color changes. The proposed strategy made the mAP attenuation amplitude of the model less than 5% on the low-light and strong-reflection test subsets, which verified the effectiveness of the proposed strategy.

The time complexity of the object recognition module’s algorithm is predominantly determined by the operations in the convolutional and fully connected layers. Given an input image of dimensions W×H, with C representing the number of input channels and $C^{\prime }$ denoting the number of output channels, and assuming the convolution kernel has an area of $k^{2}$, the time complexity of the convolution operation is

(5) $$O\left(C\times W\times H\times C^{\prime }\times k^{2}\right).$$

The detection part of YOLOv8n includes multiple convolutions and fully connected calculations, so its overall time complexity is usually a function of the image size and the number of model layers. Since the model is pruned, its time complexity is calculated according to the reduction in redundant channels. The new time complexity can be expressed as follows:

(6) $$O\left(0.6\times C\times W\times H\times C^{\prime }\times k^{2}\right).$$

#### 3.2.2. Path Planning Module

**Figure 2:**
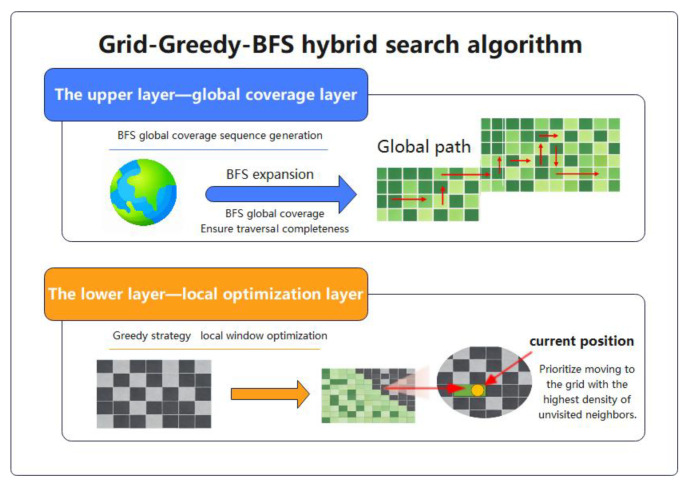
Flowchart for Path Planning Algorithm.

The path planning module proposed a grid-greedy-BFS hybrid search algorithm (GGB). The algorithm adopted a two-layer architecture: the upper layer (global coverage layer) generated a global coverage sequence based on BFS to ensure the completeness of the traversal and the lower layer (local optimization layer) optimized the subsequent selection based on the greedy strategy in the current local window and gave priority to the raster with the highest density of unvisited neighbors to reduce unnecessary backtracking, as shown in Figure 2.

The local optimum selection formula of the lower-level algorithm is as follows:

(7) $$Next\;Position=\arg\underset{v\in N(u)}{\max}\rho \left(v\right).$$

In addition, to speed up the path search and take advantage of the target location information, the A* algorithm was used to calculate the shortest path from the current point to the missing point. After each step, the A* algorithm selected the expansion node according to the formula

(8) f(n)=g(n)+h(n),

The Manhattan heuristic, $h_{\mathrm{Man}}(n),\;$ was used when the grid was four-connected:

(9) $$h_{Man}\left(n\right)=\left|x_{n}-x_{goal}\right|+\left|y_{n}-y_{goal}\right|,$$

The Euclidean heuristic, $h_{\mathrm{Euc}}(n),\;$ was chosen when diagonal motion was allowed:

(10) $$h_{Euc}\left(n\right)=\sqrt{{\left(x_{n}-x_{goal}\right)}^{2}+{\left(y_{n}-y_{goal}\right)}^{2}}.$$

At the same time, the module was gifted with an integrated mechanism for handling missed areas and dynamic replanning capabilities. Upon the initial traversal, it identified and charted the shortest route to access unvisited “void” regions. The processing of omission points was achieved through the computation of the shortest return path. Its formula was

(11) Missing Points={(x,y)|Z(x,y)=0}.

When the target recognition module found a suspected target, the system suspended the current BFS task, inserted the target point as a high-priority node, and the greedy algorithm planned a path to confirm it. After completing the task, coverage was continued from the breakpoint.

Compared with the pure BFS algorithm, the GGB algorithm reduces the invalid backtracking in the traversal process through local greedy inspiration, and the task completion time is reduced by 83.8% compared with the pure BFS algorithm under the premise of ensuring the coverage rate. Compared with the pure greedy algorithm, the GGB algorithm better avoids the omission of critical areas, and the coverage rate is increased by 16.4%.

##### Feasibility Analysis

To analyze the theoretical properties of the GGB algorithm, the search region was represented as a finite four-connected grid graph G = (V, E), where V was the set of all reachable grid nodes, E was the set of edges connecting adjacent reachable grid nodes and the starting point was denoted by s.

The GGB algorithm first used BFS to check the reachability of the connected component containing the starting point. If there was a node inside a no-fly zone that could not be reached from the starting point s, it would be indicated that there was an unreachable area in the current map and the algorithm would return an error; if the check passed, then:

∀v∈V,v was connected to s.

This meant that any traversable cell could be reached from the starting point. Therefore, the algorithm could construct a feasible path covering all traversable nodes by combining the main traversal path with the backtracking path. Consequently, if the reachability check passed, the GGB algorithm could generate a feasible path covering all traversable cells.

##### Convergence Analysis

Due to the limited size of the grid map, let the number of reachable nodes be |V| = N. During the main traversal phase, the algorithm added one unvisited node to the path at a time and marked it as visited; therefore, the set of visited nodes Vt was non-decreasing:

$$V_{t}\subseteq V_{t+1}.$$

When the new node was successfully visited at step $V_{t+1}$, then

$$\left|V_{t+1}\right|=\left|V_{t}\right|+1.$$

Since N was finite, most (N−1) new visit operations were performed during the main traversal phase. After the main traversal was completed, the algorithm obtained a set of missed nodes:

$$M=V\backslash V_{t}.$$

Since the reachability check had passed, each unvisited node was connected to the current position, and the algorithm could visit the unvisited nodes sequentially by backtracking along the shortest paths. Since the number of unvisited nodes was finite, the backtracking process would also terminate within a finite number of steps. Finally, the algorithm returned from the current position to the starting point, forming a closed path. Therefore, there existed a finite number of steps T such that

$$\exists T<\infty ,V_{T}=V.$$

That is, the GGB algorithm converged in a finite number of steps on finite-grid maps.

##### Coverage Convergence Analysis

Define the coverage at step t as

(12) $$C\left(t\right)=\frac{\left|V_{t}\right|}{\left|V\right|}.$$

During the main traversal phase, the coverage increased with each new node visited:

(13) $$C\left(t+1\right)-C\left(t\right)=\frac{1}{\left|V\right|}.$$

If the main traversal could not be further extended, the algorithm entered the backtracking phase. Since the set of missed nodes M was finite and all missed nodes were reachable, after backtracking was complete, we could conclude that

$$M=\emptyset ,\left|V_{T}\right|=|V|.$$

So

C(T)=1.

That is, provided that the reachability check passed and the backtracking process was fully executed, the theoretical coverage of the GGB algorithm converged to 100%.

##### Optimality Analysis

The GGB algorithm exhibited local optimality. During the main traversal phase, the algorithm prioritized maintaining the current direction of movement; if the adjacent nodes in the current direction were valid and unvisited, the current direction was kept; otherwise, other directions had to be tried. The strategy could be regarded as minimizing the cost of local direction changes:

min∆θ.

Here, ∆θ represented the changes in direction between two consecutive steps. For UAVs, reducing turns helped lower the cost of attitude adjustment and control, resulting in a path that closely resembled a continuous sweeping coverage path. The local selection process could be expressed as

(14) $$v_{t+1}=\arg\underset{u\in N(v_{t})}{\min}J(u).$$

Here, $N(v_{t})$ denoted the set of feasible neighbors of the current position, and J(u) was the local cost function, which could be expressed as

(15) J(u)=αΔθ(u)+βI(u).

In this equation, Δθ(u) and I(u) represented the turning cost and the node-visiting penalty term, respectively, and α and β were weight coefficients. Therefore, at the single-step decision level, the GGB algorithm prioritized feasible nodes with low local costs, thereby reducing redundant visits and frequent turns.

It should be noted that the GGB algorithm was a lightweight hybrid heuristic method designed to find high-coverage paths with low computational complexity, rather than to determine the globally shortest covering path. Therefore, while the algorithm was locally optimal and converged in a finite number of steps, it did not guarantee a globally optimal solution.

##### Overall Time Complexity of the Algorithm

Since the task environment is divided into a grid map of *M* × *N*, the BFS full-coverage time complexity should be O(M×N). The greedy algorithm selects the optimal neighbor node at each step, so for each grid node, it needs to traverse at most four neighbors (up and down, left and right). Therefore, in the worst-case scenario, the time complexity of the local optimization is O(4×M×N), indicating that each grid node undergoes at most four comparison operations. Because the overall time complexity of the GGB algorithm is a combination of the full-coverage layer (BFS) and the local optimization layer (greedy), the time complexity of the whole algorithm is O(M×N).

Furthermore, the time complexity of dynamic replanning is dependent on the number of paths requiring replanning, denoted as γ. For the computation of paths involving missed points, the A* algorithm was employed to determine the shortest path. The time complexity of the A* algorithm was expressed as $O\left(\gamma^{2}\right)$, which corresponded to the overall time complexity of dynamic replanning and missed-point processing. Putting the above parts together, the time complexity of the overall algorithm is as follows:$\;O(M\times N)+O(\gamma^{2})$. For large-scale scenes, the complexity of the algorithm is mainly determined by the raster coverage part.

## 4. Experimental Results and Analysis

### 4.1. Dataset and Training

Some experiments on the target recognition algorithm proposed in this paper were performed based on a detection subset constructed from the COCO 2017 dataset. To simulate visual interference scenarios commonly encountered in UAV search tasks, images were manually categorized according to background characteristics, including forest, urban, and grassland scenes. The final subset contained 18,287 images for training, 5000 images for validation, and 4670 images for testing. The training and testing data were approximately divided in a ratio of 7:3. For the overall testing of the UAV target search algorithm, the public dataset from MaixCAM was selected. It contained a total of 2000 640 × 640 images with 10 categories of animal identities. The first experiment used the AdamW optimizer with initial learning rate of 1 × 10^−3^, a momentum of 0.9, a weight decay of 0.0005, a batch size of 32, and 200 rounds of training. The second experiment employed the SGD optimizer with an initial learning rate of 0.01, a momentum of 0.9, a weight decay of 0.0005, a batch size of 32, and 20 training rounds.

### 4.2. Experimental Setup

**Figure 3:**
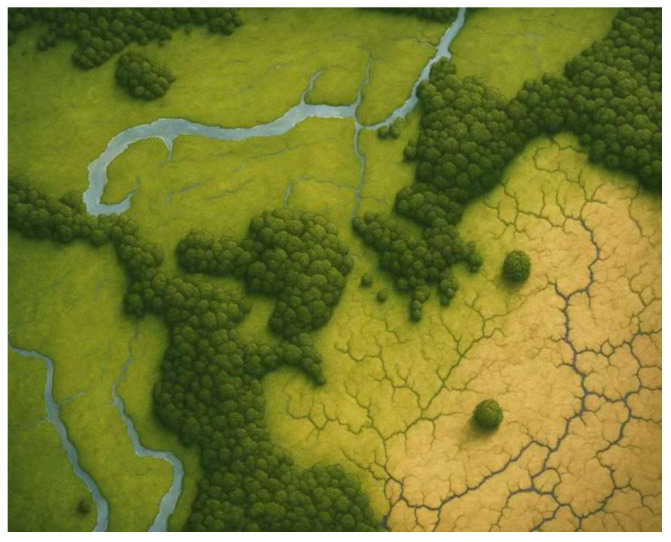
Simulation experiment scene topographic map.

The experimental hardware platform based on quadcopter and small octocopter UAVs had a MaixCAM camera in the target recognition module to perform target recognition, which was equipped with a Raspberry Pi 4B as the onboard computer to enable rapid path planning. The software environment utilizes Ubuntu 20.04 as the operating system and PyTorch 1.10 as the deep learning framework. A 30-meter-by-20-meter simulated outdoor scenario constructed indoors served as the main experimental setting, as shown in Figure 3.

**Figure 4:**
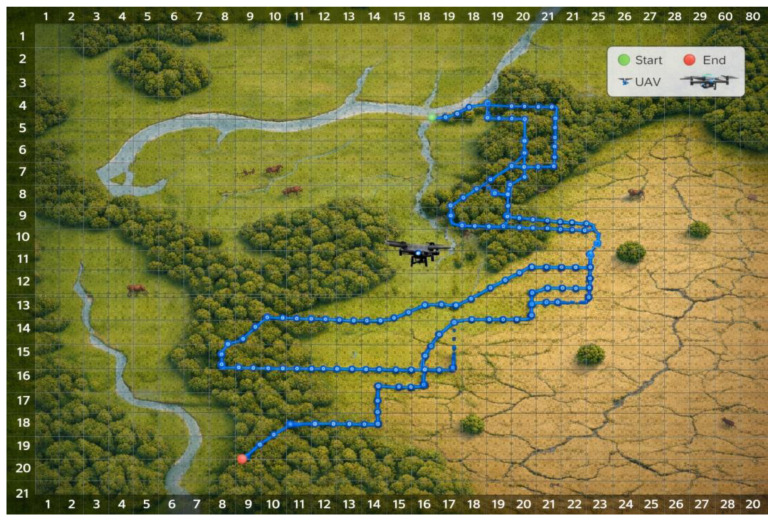
Simulation Experimental Scenario Diagram.

The experimental validation of the unmanned aerial vehicle (UAV) target search algorithm was conducted in the simulated outdoor environment described above. A total of eight targets across five distinct categories were deployed as subjects for target identification. Point A9B20 was designated as the takeoff and landing site for the UAV. Prior to the commencement of the experiment, no-fly zones were randomly established, as illustrated in Figure 4.

**Figure 5:**
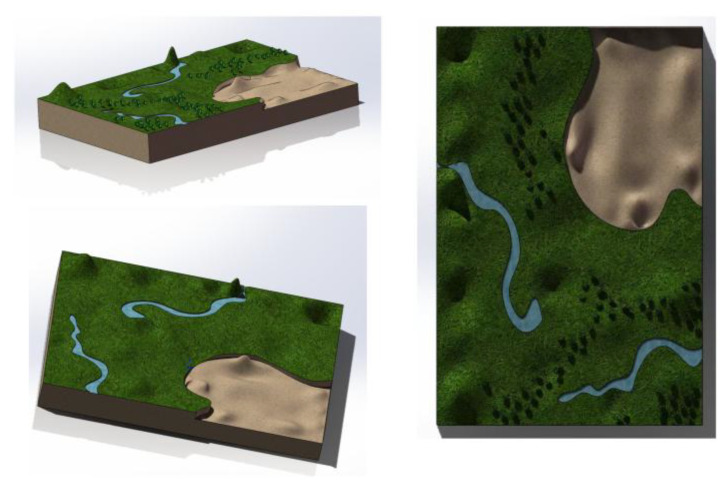
3D modeling diagram.

At the same time, three types of supplementary experiments were added to refine the experimental protocol. Several 3D simulation experiments were included as the first type of supplementary experiment, as shown in Figure 5, in which a 60 m × 40 m model of a complex open area was established using Blender and then imported into the Gazebo environment. The terrain ranged from 0 m to 8 m in elevation with slopes from 0° to 18° and involved obstacles such as trees, low walls and trellises. The environmental wind speed was set to four levels: 0, 2, 4 and 6 m/s, with gust durations of 3–8 s. The model is shown in Figure 5.

Indoor physical experiments formed the second type of supplementary experiment, in which obstacles such as foam slopes, cardboard structures and artificial tree clusters were arranged to simulate drone-blocked fields of view and no-fly zones within a 30 m × 20 m area. The selection of target objects remained consistent with that of the main experimental scenario.

Small-scale outdoor experiments formed the third type of supplementary experiment, in which low-altitude flight validation was conducted in a 50 m × 30 m area with open grassland and gently undulating terrain, with a flight altitude of 6–8 m and each mission limited to 8 min. A small octocopter drone platform equipped with a MaixCAM and a Raspberry Pi 4B was used to capture images of animal models, toy vehicles and targets covered with camouflage fabric. To ensure safety, experiments were not carried out during heavy rain or strong winds; rainy and foggy conditions were simulated by spraying water mist and applying water droplets to the exterior of the lens cover, while wind disturbances were generated by natural wind supplemented by a small handheld fan.

The parameters for all experimental scenarios in the predefined experimental protocol are shown in Table 2.

### 4.3. Performance Evaluation

In this section, the performance of the proposed algorithm is comprehensively evaluated from the three dimensions of target recognition, path planning and overall data analysis, so as to verify the effectiveness of each module and the overall framework.

#### 4.3.1. Performance Comparison of Target Recognition Algorithms

YOLOv5 and YOLOv8 are the oldest existing target recognition modules. Firstly, four small-volume models of YOLOv5 and YOLOv8 generations were compared, and it is proved that YOLOv8n was the most suitable model for deployment on a Raspberry Pi 4B. Secondly, in order to verify the effectiveness of the proposed lightweight strategy, the performance of the original YOLOv8n model and the lightweight version after channel pruning and INT8 quantization, YOLOv8N-Lite, on the Raspberry Pi 4B platform was compared on the COCO 2017 subset. At the same time, to test whether the incorporation of HSV space data provided an increased robustness effect, the attenuation amplitude mAP@0.5 was analyzed statistically on the low-illumination and strong-reflection test subset. The results are shown in Table 3.

The average inference delay of the original YOLOv8n model on Raspberry Pi 4B was about 162 ms, and the model size was 6 MB, which reached 86.2% on the training set of this paper. After pruning and quantization, the model size was compressed to 4.2 MB, the compression ratio reached 70%, the inference delay was lowered to 83 ms, representing an improvement of approximately 49% compared to the original model, mAP@0.5 was 84.6%, and the loss of accuracy was controlled within 1.6%, which met the real-time processing requirements of <100 ms on the airborne platform. After the introduction of HSV data enhancement, the mAP@0.5 attenuation of the model on the complex-illumination test subset decreased from 11.3% to 4.8%, which significantly improved the adaptability in the environment of color interference.

YOLOv8n-Lite had high detection accuracy for conventional animal models in complex background simulation experiments, while its accuracy dropped significantly for small-sized and highly camouflaged targets. The mAP@0.5 for conventional targets ranged from 72.8% to 84.6% across different test scenarios, indicating that the model possessed high real-time detection capabilities and basic target recognition performance on embedded platforms. The detection accuracy declined significantly for small-sized and highly camouflaged targets; the mAP@0.5 for these specific targets ranged from only 52.3% to 67.5%, and as the environmental complexity increased, the false negative rate rose from 6.8% in indoor simulations of outdoor scenarios to 18.6% in simulated forest-background scenarios. The detection performance in simulated forest and dense urban environments declined the most significantly, showing that factors such as complex textured backgrounds, occlusions, changes in lighting and similar colors between targets and backgrounds might undermine the robustness of visual detection models. The results are shown in Table 4.

#### 4.3.2. Performance Comparison of Path Planning Algorithms

In a 30 m × 20 m simulated field scenario, the performance of the proposed grid-greedy-BFS hybrid search algorithm was experimentally evaluated and compared with those of random walk, BFS, and greedy algorithms. The results are shown in Table 5.

The GGB algorithm achieved 98.7% area coverage in 0.31 ms, which was a 83.86% time saving compared with the pure BFS algorithm, and improved the coverage rate by 16.4% compared with the pure greedy algorithm. The algorithm also obtained a replanning delay of 28 ms, which meant it quickly responded to dynamic target confirmation tasks, taking into account both search efficiency and traversal completeness.

Further experiments compared the GGB path execution results in an ideal environment under the main experimental scenario with the results of executable paths obtained after incorporating turning radius and wind disturbance compensation in a 3D simulation scenario. In a windless environment, the algorithm achieved high coverage and mission success rates, with coverage reaching 98.7% and mission success rate at 96.0%. The average waypoint deviation and maximum deviation were 0.18 m and 0.52 m, respectively, demonstrating that under ideal or low-disturbance conditions the algorithm exhibited excellent path-tracking capabilities and area coverage performance.

However, path execution performance declined significantly under outdoor wind disturbance conditions. When wind speeds ranged from 1.5 to 2 m/s, coverage dropped to 94.3%, and the mission success rate fell to 82.0%. The mission success rate increased to 97.0% under windless conditions after incorporating motion constraints, indicating considerations on turning radii allowed the path to better align with the UAV’s actual maneuverability and contributed to reducing tracking errors caused by sharp turns or unfeasible flight segments. Subsequent introduction of wind disturbance compensation raised the coverage to 96.8% and the mission success rate to 91.0% under wind speeds of 1.5–2 m/s. Furthermore, the system coverage was 95.9% and the mission success rate was 88.0% under conditions of light rain and fog, while the average waypoint deviation and maximum deviation increased to 0.43 m and 1.11 m, respectively, showing that environmental factors such as rain and fog still exerted a certain influence on the visual localization, flight control and target identification processes. It is demonstrated that motion constraints and wind compensation strategies could effectively improve the algorithm’s feasibility and robustness in complex environments. The results are shown in Table 6.

#### 4.3.3. Serial Communication Reliability Experiment

To verify the reliability of serial communication, this experiment compared different reliable transmission mechanisms and was divided into two parts. In the vibration test, the onboard computing unit was mounted on the UAV frame and vibrations were generated by spinning the propellers and hovering at low altitude. In the electromagnetic interference test, a motor speed controller and a 2.4 GHz remote control link were placed near the communication cable harness to simulate simultaneous operation. Each group transmitted 10,000 packets of target-coordinate data. The results are summarized in Table 7.

The results showed that the optimized transmission mechanism limited the packet loss rate—caused by the combined effects of vibration and electromagnetic interference—to 0.31%. Although the average latency increased from approximately 3–4 ms for the bare serial port to 7.4 ms, it remained significantly shorter than the GGB replanning delay (28 ms) and the detection cycle (83 ms), and therefore would not become a major bottleneck in the system.

#### 4.3.4. Experiment on Raspberry Pi 4B Power Consumption and Endurance

To address the concerns regarding power consumption and flight time, the experiment used an INA219 current/voltage acquisition module and a USB-C power meter to simultaneously record the operating status of the Raspberry Pi 4B. There were four operating states involved in the experiment; a 20 min operation was carried out at each state, and the average values during the stable phases were recorded; the endurance estimate was executed based on a 3S 5200 mAh battery with an energy capacity of approximately 57.7 Wh, and the average motor power consumption during hovering and low-speed cruising was estimated at 95 W. The results are shown in Table 8.

The results showed that the lightweight model reduced Raspberry Pi 4B power consumption during continuous inference from 7.8 W to 5.1 W; after adding path planning and serial communication, the power consumption increased slightly to 5.4 W. Compared with the original YOLOv8n solution, the approach proposed in this paper prolonged the estimated system endurance by approximately 0.8 min while reducing processor temperature and the risk of thermal throttling. Since the rotor motors were still the primary power-consuming components, processor optimization had a limited impact on overall runtime, while directly affecting mission stability and real-time performance.

## 5. Discussion and Conclusions

In this paper, we propose a comprehensive algorithm framework integrating lightweight target recognition and hybrid path planning for the autonomous target search task of UAVs. Aiming at solving the problems concerning the limited resources of airborne platforms, the YOLOv8n model is compressed jointly by channel pruning and the INT8 quantization method. At the same time, an HSV spatial data enhancement strategy is introduced to significantly improve the color robustness of the model under complex lighting conditions. For the path planning aspect, a grid-greedy-BFS hybrid search algorithm is designed. Through the two-layer architecture of “global coverage + local greedy optimization”, it has the ability to provide high coverage and fast cruising, and has dynamic target replanning ability. The UAVs’ resistance to interference was enhanced by developing a lightweight and reliable transmission mechanism, so as to facilitate UAV data transmission. The experiments covered a range of common environmental conditions and the test results validated the algorithm’s effectiveness and practicality, providing technical support for autonomous UAV operation systems. The experimental results verify the effectiveness and practicability of the proposed algorithm, which can provide technical support for autonomous operating systems for UAVs.

It should also be noted that, although the algorithm has demonstrated excellent performance in indoor and simulated environments, there is a lack of test data from real-world outdoor settings with complex terrain. In view of the above limitations, we plan to further study and expand the application scenarios and generalization ability of the method in the future, so as to better meet the needs of a wide range of tasks.

## Figures and Tables

**Table 1:** Common symbols and meanings.

Symbols	Meaning
X	Input feature map
Y	Output feature map
W	Convolution kernel
$b_{k}$	Bias for the KTH kernel
i,j	Outputs the spatial position coordinates in the feature map
m,n	Internal coordinates of the convolution kernel
c	Input channel index
k	Output channel (kernel number)
C	Number of channels to input the feature map
M,N	The size of the convolution kernel
K	Number of convolution kernels (number of output channels)
S	Size of the mesh
$x_{i},y_{i}$	The center position of the target box (predicted value)
${\hat{x}}_{i},{\hat{y}}_{i}$	The center position of the true box (target value)
$w_{i},h_{i}$	Width and height of the target box (predicted value)
${\hat{w}}_{i},{\hat{h}}_{i}$	Width and height of the true box (target)
$C_{i}$	Confidence score for the target box
$p_{i}$	The probability of the target class
$\lambda_{coord},\lambda_{noobj}$	Weighting factors that modulate the effect of different terms
v	The neighborhood of the current grid
N(u)	The neighborhood of the current raster u
ρ(v)	The density value of the raster
g(n)	The known cost to the starting point n
h(n)	A heuristic estimate of cost to the goal n
Z(x,y)	Map state matrix
(x,y)	Missing point coordinates
γ	Number of dynamic obstacles

**Table 2:** Experiment scenarios and parameters.

Scenario	Dimensions	Environmental Disturbances
Indoor physical scenario	30 m × 20 m	Windless, low light and reflective
3D simulated terrain	60 m × 40 m	Artificial wind, gusts
Simulated foreign terrain	20 m × 20 m	Blocked by dense forest
Small open outdoor area	50 m × 30 m	Natural wind

**Table 3:** Performance comparison of target recognition module on Raspberry Pi 4B.

Model	Platform	Reasoning Time	mAP@0.5	Amplitude of Decay
YOLOv5nu	Raspberry Pi 4B	126 ms	54.9%	—
YOLOv5su	Raspberry Pi 4B	380 ms	68.8%	—
YOLOv8n	Raspberry Pi 4B	162 ms	86.2%	11.3
YOLOv8s	Raspberry Pi 4B	400 ms	70.8%	—
YOLOv8n-Lite	Raspberry Pi 4B	83 ms	84.6%	4.8

**Table 4:** Detection results for small or camouflaged targets in complex environments.

Test Scenario	Conventional Object mAP@0.5	Special Object mAP@0.5	Recall	Missed Detection Rate
Indoor simulation of field conditions	84.6 ± 1.2%	67.5 ± 1.8%	93.2%	6.8%
Outdoor open grassland	81.2 ± 1.5%	61.8 ± 2.1%	90.3%	9.7%
Simulated undulating terrain	78.4 ± 1.7%	57.6 ± 2.4%	87.6%	12.4%
Simulated forest background	72.8 ± 2.3%	52.3 ± 2.9%	81.4%	18.6%
Simulated densely populated urban area	74.1 ± 2.0%	55.9 ± 2.6%	84.2%	15.8%

**Table 5:** Comparison results of path planning algorithms.

Algorithms	Completion Time	Coverage	Replanning Delay
Random walk algorithm	1.15 ms	76.2%	—
BFS	1.92 ms	99.1%	45 ms
Greedy algorithm	3.06 ms	82.3%	12 ms
GGB	0.31 ms	98.7%	28 ms

**Table 6:** Path execution results under dynamic constraints and environmental disturbances.

Experimental Conditions	Coverage	Average Waypoint Deviation	Maximum Deviation	Mission Success Rate
Windless environment	98.7%	0.18 m	0.52 m	96.0%
Outdoor wind speed: 1.5–2 m/s	94.3%	0.74 m	1.86 m	82.0%
Windless environment under motion constraints	98.5%	0.14 m	0.41 m	97.0%
Wind compensation under motion constraints	96.8%	0.39 m	1.02 m	91.0%
Light rain and fog under motion constraints	95.9%	0.43 m	1.11 m	88.0%

**Table 7:** Results of comparative tests on communication reliability.

Communication Solution/Environment	Packet Loss Rate	CRC Error Rate	Average Latency	Error Times for Triggering Replanning/10,000 Packets
Bare serial port—static indoor	0.18%	0.11%	2.1 ms	7
Optimization algorithm—static indoor	0.02%	0.01%	2.8 ms	0
Bare serial port—vibration	2.73%	1.96%	3.4 ms	64
Optimization algorithm—vibration	0.11%	0.05%	5.7 ms	2
Bare serial port—electromagnetic interference	3.21%	2.44%	3.8 ms	71
Optimization algorithm—electromagnetic interference	0.16%	0.07%	6.1 ms	3
Optimization algorithm—vibration + electromagnetic interference	0.31%	0.14%	7.4 ms	5

**Table 8:** Estimated results for Raspberry Pi 4B power consumption and endurance.

Operating State	Average CPU Usage	Memory Usage	Raspberry Pi 4B Power Consumption	Temperature	Estimated Endurance
Idle system only	12%	0.9 GB	3.2 W	43.1 °C	35.2 min
Native YOLOv8n inference	76%	1.8 GB	7.8 W	66.4 °C	33.6 min
YOLOv8n-Lite inference	58%	1.3 GB	5.1 W	57.2 °C	34.5 min
YOLOv8n-Lite+GGB+communication	63%	1.4 GB	5.4 W	58.6 °C	34.4 min

## Data Availability

The original contributions presented in this study are included in the article. Further inquiries can be directed to the corresponding author.

## Abbreviations

The following abbreviations are used in this manuscript:

UAVs	Unmanned Aerial Vehicles
BFS	Breadth-First Search
CCP	Coverage Path Planning
GGB	Grid-Greedy-BFS Hybrid Search Algorithm
